# Autophagy: A Double-Edged Sword in Male Reproduction

**DOI:** 10.3390/ijms232315273

**Published:** 2022-12-03

**Authors:** Qiu Yan, Yong Zhang, Qi Wang, Ligang Yuan

**Affiliations:** 1 College of Veterinary Medicine, Gansu Agriculture University, Lanzhou 730070, China; 2 Gansu Key Laboratory of Animal Generational Physiology and Reproductive Regulation, Lanzhou 730070, China; 3 College of Life Science and Technology, Gansu Agriculture University, Lanzhou 730070, China

**Keywords:** reproduction, testes, spermatogenesis, autophagy

## Abstract

Autophagy, an evolutionarily conserved cell reprogramming mechanism, exists in all eukaryotic organisms. It is a fundamental and vital degradation/recycling pathway that removes undesirable components, such as cytoplasmic organelles, misfolded proteins, viruses, and intracellular bacteria, to provide energy and essential materials for organisms. The success of male reproduction depends on healthy testes, which are mainly composed of seminiferous tubules and mesenchyme. Seminiferous tubules are composed of Sertoli cells (SCs) and various germ cells, and the main functional part of mesenchyme are Leydig cells (LCs). In recent years, a large amount of evidence has confirmed that autophagy is active in many cellular events associated with the testes. Autophagy is not only important for testicular spermatogenesis, but is also an essential regulatory mechanism for the ectoplasmic specialization (ES) integrity of SCs, as well as for the normal function of the blood–testes barrier (BTB). At the same time, it is active in LCs and is crucial for steroid production and for maintaining testosterone levels. In this review, we expanded upon the narration regarding the composition of the testes; summarized the regulation and molecular mechanism of autophagy in SCs, germ cells, and LCs; and concluded the roles of autophagy in the process of spermatogenesis and testicular endocrinology. Through integrating the latest summaries and advances, we discuss how the role of autophagy is a double-edged sword in the testes and may provide insight for future studies and explorations on autophagy in male reproduction.

## 1. Introduction

The discovery of autophagy represented a milestone in research on human life sciences. Autophagy is a “self-eating” phenomenon in cells and is different to the “suicide” phenomenon of apoptosis [1]. Autophagy is an intracellular degradation system that relies on lysosomes to degrade cytoplasmic components, including macromolecules and organelles [2]. A growing amount of research has shown that autophagy plays an extremely important role in the male reproductive process. For example, autophagy was proven to widely participate in the regulation of spermatogenesis and in the differentiation process of spermatozoa [3,4]. Testosterone is mainly synthesized in Leydig cells (LCs) and is indispensable for male development and in maintaining sexual function [5], and autophagy contributes to testosterone production by offering certain substrates [6,7]. Interestingly, testosterone inhibits autophagy [8], with autophagy being a double-edged sword in the testicular endocrinology when an organism is exposed to endocrine-disrupting chemicals. In addition to LCs, Sertoli cells (SCs) are another vital somatic cell comprising seminiferous tubules found in testis, providing nutrition and protection for the developing spermatozoa. All stages of spermatozoa development occur on the surfaces of the SCs [8]. Another crucial function of SCs is to secrete androgen-binding protein (ABP), a testicular glycoprotein facilitating the transportation of testosterone and dihydrotestosterone [9]. A complicated study demonstrated that testosterone acts as a specific switch controller to selectively manipulate the autophagic degradation of ABP in rat SCs [8]. In addition, Ahmed N’s research provided the first clear evidence of a liability pattern for lipid consumption within SCs, demonstrating that autophagy is involved in testosterone biosynthesis and may supply endogenous energy for the development of germ cells [10]. There are data showing that stages VII-VIII of the spermatogenic cycle exhibit high levels of autophagy in SCs for the stress conditions, such as androgen receptor (AR) suppression, lipid accumulation, and mitochondrial damage. This phenomenon of high autophagy is essential for ensuring the viability of SCs and for supporting germ cells in adverse environments [11]. Similarly, several autophagy-deficient organisms produce a variety of reproductive abnormalities or sometimes cause infertility [12].

At present, the involvement of autophagy in testicular function has received a lot of attention, but there are no systematic studies reporting on autophagy in male reproduction. In this review, we consult the research progress regarding autophagy in the field of male reproductive physiology to summarize the phenomenon and the regulation of autophagy in testicular somatic cells and in various germ cells, as well as its main molecular mechanism. In addition, we also determine the occurrence and influence of autophagy on a series of events that take place during testicular development, with the aim of providing a more systematic reference for the study of testicular autophagy.

## 2. What Is Autophagy?

The essential difference between humans and machines is that our cellular homeostasis requires a dynamic balance between biosynthetic and catabolic processes [13]. Every protein has a distinct in vivo half-life time that ranges broadly from a few minutes to more than 100 days [14,15]. Eukaryotic cells primarily use two distinct mechanisms for large-scale degradation; one is proteasomes and the other is the lysosomal-dependent protein degradation pathway, namely autophagy. Only autophagy has the capacity to degrade entire organelles [16]. The discovery of autophagy was a long campaign. Primarily, lysosomes were first found by C de Duve in a consecutive centrifugation of rat liver homogenate [17]. Then, lysosome was proven to be a morphological entity via electron microscopic studies [18]. Soon afterwards, a double-membrane-bound structure containing cytoplasm and organelles was found and named “autophagosome” [19,20]. Based on all of these observations, C de Duve defined this mode of delivery of cytoplasmic materials to the lysosomes for degradation as “autophagy”, which means self-eating in Greek, in 1963 [21].

**Figure 1:**
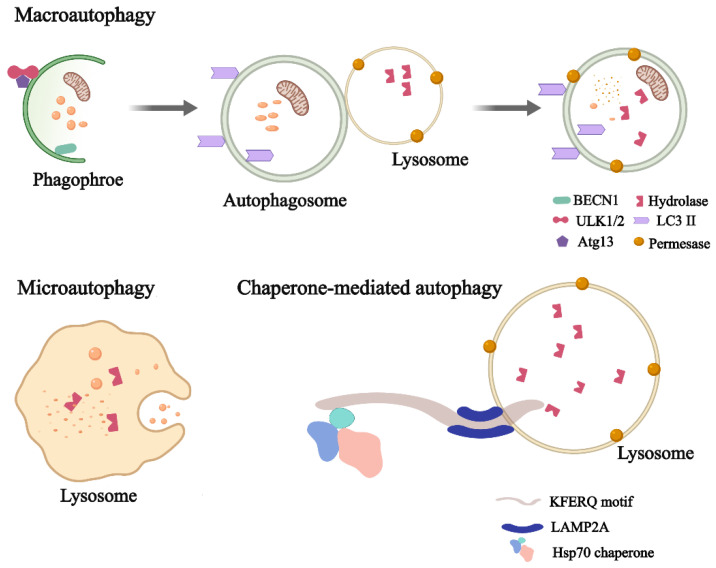
Classification of autophagy. Autophagy can be divided into macroautophagy, microautophagy, and chaperone-mediated autophagy. BECN1, beclin 1; ULK1/2, UNC-51-like kinase 1, UNC-51-like kinase 2; LC3 II, microtubule-associated protein light chain 3 II; Atg 13, autophagy-related gene 13; LAMP2A, lysosomal-associated membrane protein 2; Hsp70, heat shock protein 70.

So far, autophagy has been divided into three types, roughly according to the different molecular mechanisms: microautophagy, macroautophagy, and chaperone-mediated autophagy (Figure 1). In microautophagy, the lysosome directly engulfs the cytoplasm via the inward invagination of the lysosomal membrane. With the help of chaperone proteins, selective proteins can be targeted and translocated to the lysosomal lumen, a process known as chaperone-mediated autophagy. Macroautophagy, the most extensively studied type of autophagy, requires the use of an intermediate organelle: autophagosomes, which fuse with the lysosome to become an autolysosome and degrade the materials contained within it [22,23]. Importantly, within the last few years, compelling evidence has begun to emerge and more detailed classifications of autophagy have been reported. In brief, autophagy can also be divided into selective autophagy and nonselective autophagy based on substrate selectivity. Nonselective autophagy means that the lysosomes become flooded with degraded organelles or other harmful cytoplasmic components. While selective autophagy is easier to explain, it refers to the degradation of a specific substrate [24]. Mitophagy refers to the selective removal of mitochondria by autophagy [25,26]. When mitochondria are subjected to stimuli, such as ROS, nutrient deprivation, or hypoxia, then the damaged mitochondria are depolarized and specifically encapsulated into autophagosomes and fused with lysosomes [24,27]. Lipophagy is a new lipid degradation pathway. Lipids can be selectively degraded by the lysosomal pathway, updating our understanding of lipid metabolism [28,29]. In addition, there are also ribophagy, reticulophagy, crinophagy, zymophagy [30], pexophagy [31], and xenophagy [32,33]. Without special instructions, the autophagy processes mentioned later on in this review refer to macroautophagy. 

As a tightly regulated process, macroautophagy in mammals can be divided into five processes: induction and nucleation, elongation, closure and maturation, fusion, and degradation [34]. Firstly, autophagosome biogenesis begins with the initiating phagophore membrane, usually the endoplasmic reticulum. Then, autophagy-related gene 13, Atg13, combines with the activated UNC-51-like kinases (ULK1 and ULK2) and recruits Atg101 and the focal adhesion kinase family-interacting protein of 200 kDa (FIP200) to create a complex, representing the onset of autophagic occurrence [35,36,37,38]. The Atg14-containing class III phosphatidylinositol 3-kinase (PtdIns3K) complex needs to be recruited to the putative site of autophagosome formation in the nucleation process [39]. Subsequently, two ubiquitin-like (UBL) conjugation proteins systems: the Atg5–Atg12 conjugation system and the microtubule-associated protein light chain 3 (LC3)/Atg8 conjugation systems [33,40] play essential roles in the formation of the double membrane [41,42]. The double-membrane autophagosome sequesters the targeted substrates, so the outer membrane of the autophagosome will then fuse with the lysosomal membrane to form an autolysosome [43]. The contents of the autolysosome are then degraded and recycled by the cell.

As research deepens, it is now recognized that autophagy is a highly regulated and evolutionarily conserved cellular process that contributes to the routine turnover of cytoplasmic components. It is crucial for development, differentiation, and tissue remodeling in various organisms [1,16] and has been shown to participate in the adaptation to starvation or in the response to infection, tumor suppression, lifespan extension [44], and cell death [45]. Furthermore, autophagy has been linked to innate and adaptive immunity [46] and takes part in lipid metabolism [29], protein aggregation degradation [47], RNA degradation [4], the elimination of supernumerary or damaged organelles [16,48], ferritin degradation [49], etc.

Hence, since autophagy plays so many important roles in the development of organisms, what role does it play in the reproductive physiology of the testis?

## 3. Physiological Effects of Autophagy on Male Reproduction

High-quality spermatozoa are the key to successful reproduction in males and, in mammals, it is dependent on the healthy testis. The testis consists of somatic cells (such as LCs and SCs) and germ cells. SCs bend themselves to construct the microenvironment for the complex process of spermatogenesis [50]. LCs devote themselves to produce androgen hormones (testosterone, androstenedione, and dehydroepiandrosterone) for the development of secondary sexual characteristics and drive sexual behavior [51]. Autophagy participates in almost all of the above-mentioned processes to ensure successful male reproductive function.

### 3.1. Autophagy in Different Types of Testicular Cells

The testis is the basis of male reproduction and mainly consists of two parts: seminiferous tubules and the mesenchyme. Seminiferous tubules are composed of SCs and various germ cells, and they are the principal site of spermatozoa production. SCs are vital for supporting and nourishing germ cells and to determine the number of germ cells because they are tightly linked to the blood–testis barrier (BTB) [52]. The main functional part of mesenchyme is LCs, which are important for secreting androgens to regulate the process of spermatogenesis from the endocrine terms [52]. Autophagy performs different roles in the different types of testicular cells, and autophagy dysfunction in either of these cell types will alter the healthy development of the testis to differing degrees.

For example, the specific disruption of autophagy in mouse SCs led to ectoplasmic specialization (ES) assembly being disrupted, which resulted in disordered cytoskeleton structures. Finally, male mouse fertility manifested as spermatozoa with malformed heads being generated [53]. The main physiological roles of autophagy in other types of testicular cells are shown in Table 1.

### 3.2. Autophagy in Spermatogenesis

**Figure 2:**
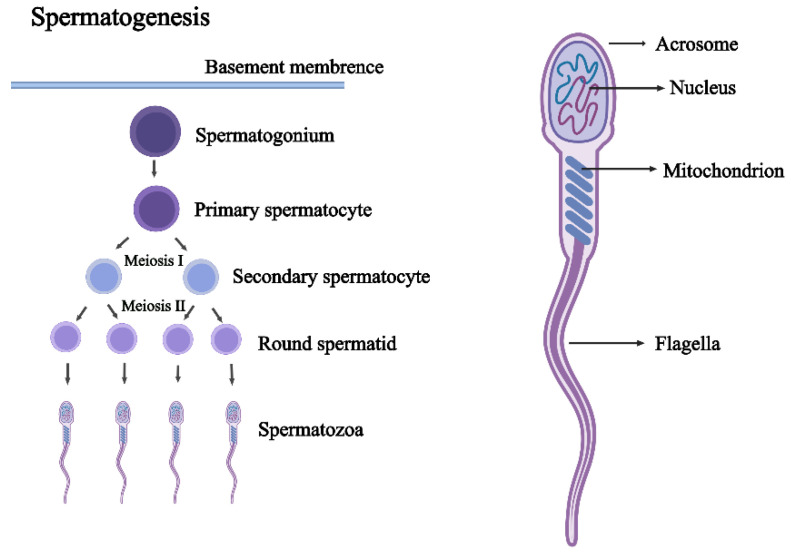
The process of spermatogenesis and the structure of spermatozoa. During spermatogenesis, each diploid primary spermatocyte develops into four haploid round spermatids through meiosis. One primary spermatocyte undergoes a round of DNA replication and meiosis I to produce two haploid secondary spermatocytes. Subsequently, cells proceed through the second cell division stage (meiosis II) to produce four haploid round spermatids. The main structure of spermatozoa consists of an acrosome, nucleus, mitochondrion, and flagella.

The development of male germ cells consists of spermatogenesis, maturation, and capacitation. Spermatogenesis is an intricate cellular process that occurs cyclically in the epithelium of the seminiferous tubule in mammalian testes. It can be further subdivided into spermatocytogenesis, spermatidogenesis, spermiogenesis, and spermiation, uniformly described as spermatogenesis in this review. It includes the self-renewal of spermatogonia via mitosis, two meiotic divisions of the spermatocytes, spermiogenesis, and spermiation (Figure 2) [58,59]. The microenvironment of spermatogenesis consists of SCs, LCs, and vascular endothelial cells.

Autophagosomes phagocytose cell components and enzymatically regenerate them into sugars, lipids, amino acids, nucleosides, and other basic nutrients to achieve intracellular nutrient recycling and energy supplementation [60,61]. Energy balance is an important feature for spermatozoa production in the testis. Dietary energy restriction results in a significantly lower testicular weight and a lower number of spermatids in the seminiferous tubules via autophagy activation [62]. Additionally, amino acid supplementation is an efficient and effective strategy to increase spermatozoa quality, depending on the activation of autophagy [63]. Increased scrotal temperature generates testicular heat stress and induces testicular autophagy, later causing spermatogenic arrest [64]. Thus, energy imbalance, hyperthermia, and hypoxia all induce autophagy during spermatogenesis, while the inhibition of autophagy often relies on chemical inhibitors.

The functional integrity of SCs is important for the ultimate success of spermatogenesis. On the one hand, the number of SCs ultimately determines the size of the testis and the number of maturable spermatozoa [65]. On the other hand, SCs form the BTB provide a unique and stable environment for the development of germ cells via their tight junction [66]. The BTB establishes the polarity of SCs, physically divides the seminiferous epithelium into basal and apical compartments, and is pivotal to spermatogenesis [67].

ES is a testis-specific, actin-based hybrid anchoring and tight junction and that includes basal ES and apical ES. Basal ES constructs the BTB and links it to the actin cytoskeleton [68], while apical ES is important for the development and maturation of spermatids [53,69]. Concretely, apical ES is localized at the contact surface between the Sertoli cell and the spermatids, and it is tightly connected to the sperm head via the acrosome, being an active participant in spermatozoa head shaping [40]. Based on the features of anchoring and tight junctions, ES provides effective and dynamic adhesion for developing spermatids, with spermatids being able to “anchor” onto SCs in the epithelium. It ensures the proper orientation and migration of spermatids in the seminiferous epithelium during spermatogenesis [68].

The cytoskeleton is the major mechanical structure of the cells and is a complex, dynamic, and biopolymer network that contains two major systems: microtubules and F-actin [70]. In autophagy-deficient Atg7^−/−^ mice, the F-actin was disorganized [71]. Coincidentally, when particular key proteins required for autophagy initiation were knocked out—Atg5 and Atg7—in the SCs of mice, both apical and basal ES were disrupted, and the cytoskeleton structure was disorganized, resulting in spermatozoa with malformed heads and depressed motility. This resulted in the deficiency of autophagy, with the affects being the unsuccessful degradation of PDZ and LIM domain protein 1 (PDLIM1). The accumulated PDLIM1 led to the ineffective removal of cytoplasm during spermatogenesis, disassembling the cytoskeletal components of spermatozoa [53]. Eventually, the flagellar structure in the spermatozoa was destroyed and the motion parameter of the spermatozoa changed.

In addition to being expressed in SCs, Atg7 was expressed in all types of mouse spermatogenic cells after heat treatment. At this time, autophagy, as a partner of apoptosis to induce cell death, is likely to have participated in acrosome biogenesis and in the acrosome reaction [4,72]. What is an acrosome? It is a highly evolutionarily conserved lysosome-related membranous organelle with a cap-like structure that is located in the anterior part of the spermatozoa nucleus [73]. Acrosomes are derived from the Golgi apparatus and carry hydrolytic enzymes to facilitate spermatozoa in penetrating the zona pellucida to fuse with oocytes [74,75]. Some studies have confirmed that TBC1 domain family member 20 (TBC1D20) may be regulated through the Golgi apparatus to mediate testicular function [76,77,78]. Additionally, TBC1D20 regulates the formation of acrosomes via facilitating autophagy flux [79]. Furthermore, Sirt1 regulates acrosome biogenesis by modulating autophagy flux during spermiogenesis [80].

When it comes to acrosome biogenesis, we have to think about flagella biogenesis, as both are the most important biological processes in the formation of spermatozoa. Intraflagellar transport 20 (IFT20) is a Golgi transport protein that interacts with sperm flagellar 2 (SPEF2) to co-ordinate the development of the sperm flagella, something that is reflected specifically in sperm tail formation and head reshaping [81]. Significantly, IFT20 contributes to autophagosome formation by delivering Atg16L [82], that is to say that autophagy participates in acrosome biogenesis, flagella assembly, and in the shaping of the sperm head [83]; therefore, once autophagy is disrupted or absent, it ultimately leads to structural defects in spermatozoa, abnormal spermatozoa, and even infertility. 

During spermatogenesis, each diploid primary spermatocyte (PSC) develops into four haploid round spermatids through meiosis, which occupies an absolute nuclear position in the process of spermatogenesis [84]. For haploid round spermatids, chromatoid bodies (CBs) are a typical cytoplasmic feature: unique ribonucleoprotein (RNP) granules [85,86]. Interestingly, both the agonists and antagonists of autophagy aggravate the cellular defects of haploid round spermatids, and these defects manifest as the fragments of CBs, manifesting that autophagy is involved in the clearance of CB materials and the maintenance of CB homeostasis synchronously, evidence that it is a double-edged sword [72,83]. In recent years, the interactions between autophagy-related proteins and meiosis have been proposed gradually [87]. For example, interactions between meiosis and autophagy-related proteins, such as Atg5, Atg7, Atg16, LC3, Beclin 1, p62, m-TOR, AMPKα 1/2, and PINK1, and their upstream regulators present in human spermatozoa have been observed, with autophagy activation inducing a significant increase in motility [88]. Markedly, the expression of LC3 and Atg7 is increased dramatically from round to elongated spermatids [89]. Significantly, spermatozoa are a type of highly differentiated cells that can be eliminated within SCs by autophagy in vivo, which guarantees the initiation of the next reproductive cycle [90].

### 3.3. Autophagy in the Endocrinology of Testis

**Figure 3:**
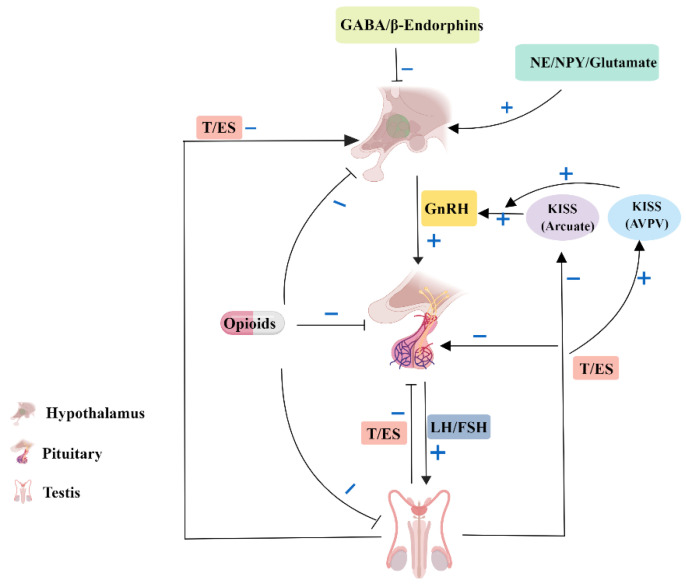
The regulation of the hypothalamic–pituitary–testicular axis. T, testosterone; ES, estradiol; NE, norepinephrine; NPY, neuropeptide; GABA, gamma-aminobutyric acid; GnRH, gonadotropin-releasing hormone; LH, luteinizing hormone; FSH, follicle-stimulating hormone; KISS, kisspeptin; AVPV, anteroventral periventricular nucleus.

Male reproduction and development, as well as the maintenance of male sexual characteristics, are principally governed by the hypothalamic–pituitary–testicular (HPT) axis. Gonadotropin-releasing hormone (GnRH) is the central regulator of the HPT axis. It is secreted by the hypothalamus and regulates the synthesis and secretion of luteinizing hormone (LH) and follicle-stimulating hormone (FSH) from the pituitary gland. LH and FSH act on the testes to stimulate the synthesis of sex gonadal steroid hormones and modulate testicular-specific morphological changes and functions [91,92,93]. Conversely, gonadal steroids provide continuous negative feedback to the hypothalamus and pituitary gland to maintain a steady state of the HPT axis and to ultimately maintain healthy male reproductive function (Figure 3) [94]. Testosterone is regulated by LH secretion. It is an indispensable hormone for sexual development and for maintaining male characteristics [95,96]. Testosterone is mainly synthesized in LCs, where autophagy has been reported to be extremely active [54,97]. The process by which LCs synthesize and secrete testosterone is susceptible to external disruptors, such as hypoxia, toxicants, drugs, and many environmental hormones, all of which can adversely affect the function of LCs and result in testosterone disorders [7]; however, these adverse factors can easily prevent the occurrence and development of autophagy. In rat LCs, abundant autophagosomes, phagocytic organelles, were observed [98]. 

Recent studies indicated that autophagy has played a vital role in the regulation of testosterone synthesis. Gao et al. [6] specifically disrupted autophagy via the conditional knockout of Atg7 and Atg5 in mouse LCs and found that there was a sharp reduction in testosterone in the serum because the disruption of autophagy interrupted cholesterol ingestion, which is similar to the symptoms of late-onset hypogonadism (LOH). LOH is a common clinical and biochemical syndrome associated with androgen deficiency, something that is primarily characterized by erectile dysfunction [99]. Further investigations revealed that interrupting autophagic flux leads to the accumulation of solute carrier family 9 (sodium/hydrogen exchanger) and member 3 regulator 2 (SLC9A3R2/NHERF2) in LCs, which results in the downregulation of scavenger receptor class B, member 1 (SCARB1/SR-BI). Ultimately, the supply of cholesterol is insufficient [6]. These results lead to speculation that the autophagy of LCs regulates cholesterol allocation for the production of androgen hormones and further regulates spermatogenesis [100], further illustrating that an imbalance in testicular homeostasis is associated with autophagy deficiencies.

Furthermore, with aging, the capacity that LCs have to produce testosterone and stimulate LH declines significantly [101], and these decreases are associated with the reductions in cyclic adenosine monophosphate (cAMP) and steroidogenic acute regulatory (StAR) [102]. In aged LCs, Li et al. [103] observed that the levels of StAR and testosterone production were lower than in young cells, which was affected by autophagic activity. This phenomenon is possibly the result of elevating the cellular ROS level. After binding StAR, free cholesterol (FC) is transferred to the mitochondria. FC is the substrate for testosterone synthesis, and its generation needs the participation of autophagy. A previous study demonstrated that inhibiting autophagy in primary rat LCs with chloroquine (CQ) or siAtg7 reduced testosterone production and decreased the level of FC [7]; here, the type of autophagy was lipophagy.

In addition to LCs, SCs are also meritorious to testicular endocrinology. Androgen-binding protein (ABP), a testicular glycoprotein secreted by the SCs, is known to bind, transport, and concentrate testosterone and dihydrotestosterone, as well as protect them from catabolism in the testicular fluids [104]. ABP is concentrated in the apical part of the SCs [105], promotes germ cell differentiation, and regulates spermatogenesis spatially [9]. Its expression level is positively correlated with spermatozoa motility [106]. Some studies have shown that the varicocele-induced dysregulation of ABP may be a parameter of impaired reproductive function [107], and testosterone is the dominant regulator of its synthesis in vivo [108]. In a detailed study, both in vitro and in vivo experiments demonstrated that autophagy regulates ABP expression. This autophagic degradation process was selectively regulated by testosterone, which prolongs ABP’s biological half-life by inhibiting autophagy [8]. More precisely, the autophagic degradation of ABP is only effective at the protein level. Interestingly, autophagy is also affected by the concentration of testosterone [54,109]. On the whole, testosterone may act through a negative feedback loop regarding autophagy to sustain cellular homeostasis, while autophagy participating in testosterone production and ABP metabolism regulates the process of spermatogenesis indirectly [40].

Moreover, this review cannot fail to mention that endogenous estrogen signaling is essential for male reproduction [110]. LCs can synthesize the estrogen, germ cells, and epididymal spermatozoa-expressed P450 aromatase (CYP19A1) and can actively synthesize estrogens from androgens as well [111]. Further studies have revealed that estrogen’s main receptor—estrogen receptor 1 (ESR1), is essential for male fertility and for the development of efferent ductules, the epididymis, and prostate and ensuring that loss of only the membrane fraction of ESR1 is sufficient to induce extensive male reproductive abnormalities and infertility [112,113].

### 3.4. The Role of mTORC1 in Autophagy in Male Reproduction

Autophagy can be triggered by a variety of internal or external stimuli through single or multiple signal pathways. Additionally, unfavorable circumstances, such as hypoxia, UV, starvation, ROS, and the accumulation of unfolded proteins, can also provoke autophagy to become a cytoprotective mechanism [114]. Generally, autophagy induced by multiple factors is more common. There are some studies that have indicated that long-term heavy metal exposure to metals, such as arsenic (As), lead (Pb), copper (Cu), and cadmium (Cd), can promote testicular apoptosis and autophagy by mediating oxidative stress, which is considered to be the key mechanism causing testicular degeneration as well as dysfunction. Additionally, drugs with antioxidant activity, such as vitamin C, can be effective in improving these testicular injuries [115,116,117].

The mammalian target of rapamycin (mTOR), a serine/threonine kinase, is a major regulator of cell growth, survival, metabolism, and immunity. It plays a central regulatory hub role in cell metabolism. As research has become more in-depth, increasing studies have shown that diverse environmental toxicants induce testicular injury and regulate autophagy via mTOR signaling [118,119]. For instance, Liu et al. [120] demonstrated that rapamycin inhibits spermatogenesis through suppressing the phosphorylation of p70S6K and changing the autophagy status, ultimately reducing the number of spermatozoa. Similarly, Xu et al. [121] observed that, upon mTOR inactivation by rapamycin, the number of spermatozoa significantly decreased and spermatogonia proliferation was blocked. 

In more detail, mTOR forms two distinct signaling complexes, mTOR complex 1 (mTORC1) and mTORC2 [122]. Noticeably, mTORC1 is the main gateway to autophagy, connecting cellular nutrient sensing with environmental cues to preserve cellular homoeostasis. Not only does mTORC1 promote cell growth by stimulating biosynthetic pathways, it also inhibits cellular catabolism through repressing the autophagic pathway [123]. Conditions of autophagy activation, such as nutrient or growth factor deprivation and low cellular energy levels, have been shown to inhibit mTORC1 activity, validating the existence of a tight inverse coupling between autophagy induction and mTORC1 activation. In other words, high mTORC1 activity promotes biomolecular synthesis and simultaneously inhibits autophagy [124,125]. Retinoic acid (RA) is required for the self-renewal of spermatogonial stem cells (SSCs) and for subsequent entry into meiosis [126]. As a central modulator in stem cell homeostasis, mTORC1 signaling governs stem cells quiescence [127,128]. The inhibition of mTORC1 blocks the RA-induced translational activation of mRNAs, resulting in an accumulation of undifferentiated progenitor spermatogonia. This imbalance between self-renewal and differentiation eventually leads to spermatogenesis defects, some of which may even result in infertility [129]. Even when knocking out the regulatory associated protein of mTOR, complex 1 (RPTOR) in the SSCs of mice, due to the absence of self-renewing spermatogonia, these mice can survive and be healthy but have smaller testes than their littermate controls, with no spermatozoa being present in their cauda epididymides [130]. These results suggest that mTORC1 is autonomously required for SSC proliferation and differentiation and that it is necessary for the development of male reproduction.

Another study discovered that the regulation of Sertoli cell proliferation by follicle-stimulating hormone (FSH) depends on the PI3K/AKT/mTORC1 pathway, while the activation of AMPK causes a decrease in mTORC1 signaling [131]. The loss of Raptor in SCs causes severe tubular degeneration in neonatal testis, and adult mice displayed azoospermia. Additionally, Raptor independently controlled cytoskeletal homeostasis and cell polarity in SCs [132]. 3-methyladenine (3-MA) is known as an autophagy inhibitor that inhibits the PI3K pathway in vitro [133]. Previous research demonstrated that 3-MA rescues apoptosis by partially aggravating the reduction in the autophagy flux in cadmium-treated mouse spermatogonia and rescued apoptosis by inhibiting autophagy in spermatocyte cells [134]. Thus, autophagy exerts different effects on spermatogonial cells and spermatocyte cells in response to external stimuli and is not a pure protective function. 

## 4. Discussion

**Figure 4:**
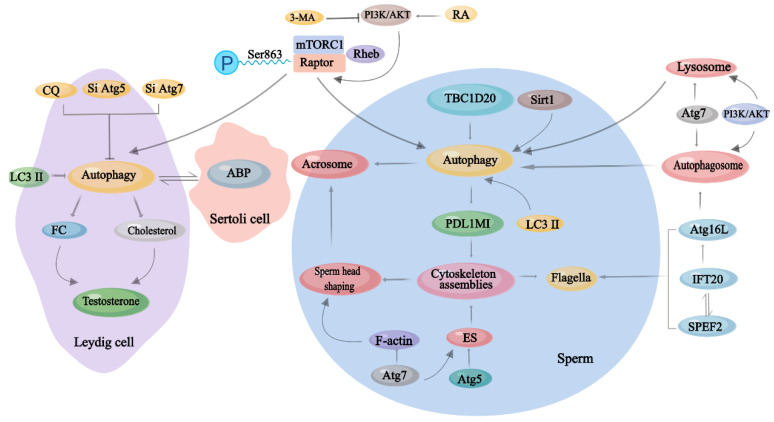
The role of autophagy in spermatogenesis. CQ, chloroquine; FC, free cholesterol; ABP, androgen-binding protein; 3-MA, 3-methyladenine; PI3K/AKT, phosphatidylinositol 3-hydroxykinase/protein kinase B; RA, retinoic acid; mTORC1, mTOR Complex 1; TBC1D20, TBC1 domain family member 20; PDL1M1, programmed cell death 1 ligand 1; ES, ectoplasmic specialization; IFT20, intraflagellar transport 20; SPEF2, sperm flagellar 2.

Autophagy is an important lysosomal pathway that removes damaged macromolecules and organelles. Cumulative results have revealed that autophagy is involved in the life processes of multiple cells within the male reproductive system and that it is active in key pathophysiological processes in many diseases of the male reproductive system, such as azoospermia, oligospermia, asthenospermia, cryptorchidism, and orchitis [40]. Autophagy can maintain the survival of testicular cells or accelerate the apoptosis of some cells, representing a double-edged sword [135]. Herein, we presented a comprehensive overview on the effects of the regulation of autophagy on male reproduction, including its relation to spermatogenesis, the endocrinology of testis, and the key molecule of autophagy mechanism—the regulation of mTORC1 (Figure 4). Before concluding, we would like to thank all of the studies cited in this review for their great contributions to opening the prologue and determining the close connection between autophagy and male reproduction and for illuminating the way forward for subsequent related studies.

Spermatogenesis, the most momentous physiological process in the male reproductive system, is a dynamic and complex process [136]. Dynamic refers to how normal spermatogenesis requires cellular homeostasis between the degradation of cytoplasmic components and the energy supply to boost these orchestrated physiological processes. The complex is reflected in this biological process, which involves the mitosis of the spermatogonia, the meiosis of the spermatocytes and spermatogenesis, and, finally, the transformation of circular spermatids into elongated spermatids [137]. Suitably, autophagy is a master of degradation and recycling, especially in the disposal of residual cell bodies and structural reconstruction [138]. There are a range of results that provide us with clues to the association among the downregulated expression of Atgs and low spermatozoa quality [139]. During spermatozoa differentiation, the expression of autophagy-related proteins, such as LC3 and Atg7, was significantly higher in elongated spermatozoa [140]. The elimination of autophagy by the germline-specific knockout of Atg7 resulted in reduced testicular weight and spermatozoa malformations, as well as significantly reducing fertility in male mice [141]. In addition to being involved in the induction of diploid germ cells and the differentiation of spermatozoa, autophagy is also involved in the structural formation of flagella and acrosomes [56]. After knocking out Atg7, the multiple vesicles of Golgi spermatozoa are unable to fuse with each other, thus exhibiting multiple acrosomal vesicle structures, and the accumulation of multiple lysosomal vesicles or aggregates from the Golgi apparatus leads to acrosomal contraction, resulting in acrosomal malformations [140]. SCs play a nursing role as structural and functional supports during spermatogenesis, ensuring the production of highly specialized mature spermatozoa [142]. Autophagy is active in SCs during spermatogenesis, and the level of autophagy becomes more evident as spermatogenesis advances from the basal to the luminal compartment of SCs [143]. Sertoli–germ cell communication is vital for germ cell development and maturity, and the lack of autophagy in SCs aggravates cadmium (Cd)-triggered apoptosis in germ cells [144]. However, autophagy is not always advantageous for spermatogenesis. Jing Yang’s group investigated whether inhibiting excessive autophagy could protect against high-fat diet (HFD)-induced spermatogenesis deficiencies and male infertility [145]. Consequently, deciphering the mechanism of action of autophagy in spermatogenesis requires us to think multi-dimensionally. In addition, autophagy is a perfect cleaner to remove spermatozoa mitochondria DNA (mt DNA) after and before fertilization [146].

Moreover, autophagy can directly affect testicular endocrine regulation via regulating the biosynthesis of testosterone [147]. Autophagy occurs in LCs hyperactively, primarily acting on the process of steroid production. A deficiency in serum testosterone levels is associated with primary or late-onset hypogonadism [148,149], which is associated with male sexual dysfunction and decreased reproductive capacity [150,151]. Because autophagy provides cells with sources of triglycerides (TGs) and cholesterol, some researchers have speculated that autophagy might be involved in testosterone synthesis by promoting lipid metabolism in LCs [6]. Thus, autophagy participates in testosterone production by providing SR-BI, the receptor of high-density lipoproteins (HDLs), to promote the selective uptake of lipoproteins [152]. The lack of autophagy in mouse LCs leads to a deficiency in cholesterol uptake and, eventually, to a decline in testosterone biosynthesis [6]. Thus, misfolding and increased aggregation in autophagy-deficient endocrine cells may be a direct result of disturbed hormone levels, which could provide new insights into addressing defects in the male reproductive system. N6-methyladenosine (m^6^A) is the most prevalent internal modification in mRNA [153]. As the research continues, there are studies proving that m^6^A mRNA methylation regulates testosterone synthesis through modulating autophagy negatively in LCs via reducing AMPK activity [154]. This provides a novel therapeutic strategy to target m^6^A RNA methylation for the treatment of azoospermia and oligospermia in patients with reduced serum testosterone [154].

mTOR is an evolutionarily conserved kinase that consists of mTORC1 and mTORC2 that is defined by the presence of the key accessory proteins Raptor and Rictor, respectively [155]. Significantly, mTORC1 governs autophagy at the lysosomal surface [156]. Uropathogenic Escherichia coli (UPEC) can cause defects in the BTB of rat testes, and the mTORC1 inhibitor rapamycin is able to significantly restore the expression of cell–junction proteins and can exert a protective effect on the BTB [157]. In the above, we highlighted the role of mTORC1 in male reproduction due to its close association with autophagy. Additionally, the role of mTORC2 in spermatogenesis cannot be underestimated. Specially, there are two ESs in the seminiferous epithelium that are dependent on location. The one near the basement membrane between adjacent SCs, localized at the BTB, is the basal ES and is responsible for Sertoli cell–cell adhesion [158]. The other one localizes to the apical compartment, the apical ES, which is the only anchoring device between SCs and spermatids [159]. During the seminiferous epithelial cycle of spermatogenesis, mTORC1 and mTORC2 exert their antagonistic effects: mTORC1 promotes BTB disassembly and mTORC2 contributes to the assembly of a “new” barrier. The delicate mTORC1–mTORC2 balance is critical to preserving the structural and functional integrity of the BTB [160,161]. However, the specific proteins involved in these dynamic events remain to be identified and examined, and a large amount of work is needed to explore how mTOR complexes exert their effects on male reproduction.

In conclusion, the review presents the double-edged characteristics of autophagy in the most important processes involved in male reproduction. Autophagy is active in many aspects of male spermatogenic and endocrinological processes. On the one hand, it is involved in the regulation of testosterone production by offering materials; consequently, autophagy inhibition drugs, such as chloroquine (CQ), should be used cautiously in patients with reproductive demands. On the other hand, testosterone inhibits autophagy in a negative feedback loop. Despite the cumulative gains revealed, autophagy is blossoming in many aspects of male reproduction. This is just the tip of the iceberg, and there are still many gaps between autophagy and male reproduction that are worthy of exploration. So far, it is unknown whether or how autophagy is involved in spermatozoa capacitation, and perhaps it represents a good potential target for exploring the molecular mechanisms in capacitation disruption-induced male infertility [83]. Endocrine-disrupting chemicals (EDCs), such as zearalenone (ZEA), can impair male reproductive health by disturbing the level of endogenous hormones and spermatogenesis processes, either directly or indirectly [162]. In rat LCs, autophagy was achieved against ZEA-induced apoptosis by reducing cytotoxicity [163]. Additionally, similar protective autophagy processes can be concluded from the dibutyl phthalate (DBP)-treated prepubertal rat germ cells [164]. A cytotoxic role of autophagy could be observed in di-2-ethylhexyl phthalate (DEHP)-treated mouse LCs and microcystin-LR (MC-LR)-treated rat SCs [165,166]. Thus, it is necessary to explore how autophagy exerts both pro-death and pro-survival double-sided effects in EDC-induced spermatozoa injury. In addition, it is also necessary to determine whether the increased vulnerability of toxic protein aggregation in endocrine cells connects with deficiencies in autophagy directly. Furthermore, why does autophagy act differently in elongated sperms than in other types of round spermatids?

Above all, based on the double-edged features of autophagy, dialectically and comprehensively exploring the specific role of autophagy in male reproduction would be useful in diagnosing and providing novel therapeutic strategies for diseases of the male reproductive system.

## Figures and Tables

**Table 1:** Physiological roles of autophagy in testicular cells.

Cell Types	Role of Autophagy	Dysfunction	References
Sertoli cells	clear ABP; regulate secretory activity; degrade useless components in seminiferous tubules; modulate ES formation; maintain the normal cytoskeletal organization	Prolongation of the half-life of ABP; disorders of the cytoskeleton of Sertoli cells; destruction of the ES structure	[8,53,54,55]
Spermatozoa	participate in acrosome biogenesis; regulate the formation of flagellum	malformation of spermatozoa head; decreasing of spermatozoa motility	[4,56]
Spermatogonial cells and Spermatogonial stem cells	adaptive protection	decreased ATP content; cell death	[3]
Leydig cells	maintain the production of testosterone; facilitate the uptake of cholesterol	steroidogenic decline	[6,52,57]

## Data Availability

Not applicable.
